# Mental Health of Dubai Medical College Students

**Published:** 2012

**Authors:** Jamshid Ahmadi, Mohammed Galal Ahmed, Fatehia Ali Bayoumi, Abeer Abdul Moneenum, Haya Alshawa

**Affiliations:** 1 Department of Psychiatry, Shiraz University of Medical Sciences, Shiraz, Iran.; 2 Dubai Medical College for Girls, Dubai, United Arab Emirates.

**Keywords:** General Health Questionnaire, Medical Students, Psychiatric distress

## Abstract

**Objective:** Considering the association between medical school dropout and psychiatric distress, we aimed to assess the prevalence of psychiatric distress among medical students at Dubai Medical College.

**Methods:** One hundred and three medical students were chosen randomly and were assessed by the General Health Questionnaire (GHQ).

** Results:** The mean age for the students was 18.85 year (Minimum: 17, Maximum: 22), and 90.3% were between 18 and 20 years old. The mean of GHQ score was 16.46. Of the participants, 47 (45.6%) were found to be in normal range (GHQ mean < 16). A total of 33 (32.1%) of the students reported evidence of psychiatric distress. Only 23 (22.3%) were found to have severe psychiatric distress.

**Conclusions: **Early detection of psychiatric distress is important to prevent psychiatric morbidity and its unwanted effects on medical students and young doctors. Our results reveals that although a low percentage of Dubai Medical College students reported a significant level of psychiatric distress, however, it should not be underestimated, and actions should be taken to encourage Dubai Medical College students to get help from for psychiatric services for their emotional problems. The risk factors as well as the protective factors must be identified in nation-wide studies to promote mental health of medical students.

## Introduction

The World Health Organization stated that the next two decades might witness worldwide changes in the pattern of epidemiology of diseases. Non-communicable diseases such as mental disorders may replace communicable diseases as the leading factor in disability and premature death (1).

Studies of disease burden have revealed the importance of psychiatric disorders. For example, depression was the fourth leading cause of disease burden, accounting for 4.4% of total disability adjusted life years in the world (2).

Higher education has always been regarded as highly stressful. Even though only the academically-minded of the population in society is eligible for higher education, this stressful environment can exert a negative effect on the psychological and physical well being of the students. This will finally result in poor academic achievements and possibly a large number of psychological casualties. Medicine has always been regarded as a popular choice in higher education. As a result of an excess of applicants, only candidates with excellent academic attainment can successfully enter medicine. Therefore, the medical program is even more competitive and stressful for students who are accepted (3-12).

A study in Singaporean using the General Health Questionnaire (GHQ) indicated that 57% of medical students had psychiatric distress compared to 47.3% of law students (7). An American study conducted in medical school at the University of Mississippi showed that of the medical students 23% had depression and 57% had high levels of emotional distress (5).

Psychiatric disorders were often reported in students reading for examinations. Other causes of psychiatric disorders were fear of failure, uncertainty regarding supervisors' expectations and uncertainties regarding achievement. Undergraduate medical students compared to any other undergraduate course have been the most distressed group of students. There have also been reports of significant psychiatric morbidity in young doctors (13, 14). 

Most people with psychiatric disorders can be successfully treated because of the existence of effective psychological and pharmacological treatments. Although more than half of all patients with emotional disorders are initially seen in the general medical system, their symptoms are frequently not diagnosed and thus they are not likely to receive proper treatment. Studies on psychiatric disorders among medical students have found that these disorders are under diagnosed and undertreated. Failure to detect these disorders will unfortunately lead to increase psychological morbidity with unwanted effects throughout their careers and lives (13).

Early detection decreases the duration of an episode of psychiatric disorder and results in far less social impairment in the long term (15).Therefore, psychiatric disorders must be managed at an early stage for a better quality of life among medical students.

The most commonly used and established screening questionnaire for psychiatric distress is the General Health Questionnaire (GHQ) which is available in many versions as short as 12 items (GHQ-12) and as long as 60 items (GHQ-60). The GHQ has been widely used in studies in many countries both in the community as well as in general practice. It is used to detect non-psychotic psychiatric disorders, such as depression and anxiety (16,17) . Its validity is well established and has high sensitivity and specificity (17,18). The short version of this questionnaire (GHQ-12) makes it easier for use in busy medical students as it takes a short time to complete the questionnaire. It is also short, simple and easy to understand.

To our knowledge, there is not any published study on psychiatric disorders among medical students in Dubai, United Arab Emirates. Therefore, it is of interest to evaluate the prevalence of psychiatric disorders in this population.

## Materials and methods

The participants were 103 pre-clinical medical students selected by random cluster sampling, from International Dubai Medical College for Girls who were administered GHQ-12 in 2007.

The reasons behind choosing a multiple-choice questionnaire (GHQ-12) are to limit the responding time, and to elicit more specific and objective answers. 

The questionnaires were distributed, completed by the students, and collected in the same session. Distribution was followed by a full explanation of the reasons for the implementation of the study and the students were informed that their responses would be confidential. They were asked to write their personal comments and recommendations at the end of the questionnaire. Special attention was paid to ensure that the students clearly understand the instructions about answering the questionnaire. In addition they were asked not to write their name or student number on the questionnaire in order to encourage them to provide more open and honest answers. The students were given enough time to complete and return the GHQ-12. 

The participants' answers were scored as 0-1-2-3 based on their responses. The total score was determined by adding the score obtained for each answer in the questionnaire. Based on the GHQ-12 guidelines, scores of 12 and above were considered to be positive for psychiatric distress (16).

## Results

 All of the students completed the questionnaires. Their mean age was 18.85 years (SD, 0.99). (Minimum: 17, Maximum: 22). The majority 93 (90.30%) were between 18 and 20 years and only 7 (6.80%) were older than 20 years. The mean score of the GHQ for the students was 16.46, (SD, 5.69). Of the participants, 47 (45.6%) were found to be in normal range (the GHQ mean < 16). A total of 33 (32.1%) of the students reported evidence of psychiatric distress. Only 23 (22.3%) were found to have severe psychiatric distress*.*

## Discussion

 Psychiatric distress has commonly been found among medical students. The prevalence of psychiatric disorders were reported to be higher in students who complained of pressure of exams, as well as students who did not have a good relationship with their parents, siblings and teachers (9). It is very important to detect psychiatric distress at an early phase so that treatment in the form of counseling, behavior therapy, cognitive therapy and even pharmacotherapy can be considered for those affected. Therefore, psychiatric morbidity such as depression and anxiety can be prevented among our medical students and young doctors (19).

Medical program is always very competitive and stressful. Medicine has been considered as a popular choice in higher education. As a result of an excess of candidates, only those with high academic performance can be accepted and enter into medical school (7,14).

Our study showed that the overall prevalence of psychiatric distress among medical students was much lower compared to other studies from Singapore (7) and the USA (5). A study on stress, using the GHQ, among students in Singapore showed that the prevalence of psychiatric distress was 57% in medical students (7). Mosley et al. reported that 57% of the third year medical students complained of psychological distress (5).

Firth-Cozens indicated that clinically significant levels of psychiatric distress were often reported in medical students who were studying for exams (13). The distress was often as a result of fear of failing their exams. Students who had uncertainties regarding their supervisors' expectations and their own performances in examinations were more prone to developing psychiatric distress (13) . Ko *et al*. reported that at least 70% of medical students complained of stress related to academic performance (7). They also found that students who had good relationships with their parents and siblings were able to cope with their problems. These students reported that their families were an important source of support to them; the parents as a source of financial support, while siblings serve as confidantes for psychological problems (7). Many medical students are also able to cope with their problems by sharing with their classmates. When faced with a problem, they turn to their friends and classmates for assistance (7).

Most of the studies have indicated that psychiatric problems are more common among females than males (15,18,20).

In a study factors found to be associated with psychiatric disorders among medical students in Singapore were adjusting to different environments, increased academic pressure and little time for personal activities (7)^.^ The students’ experience of today is very different to that experienced in the 1960s, 1970s or 1980s. Recent research in the UK indicates that mental health or psychological problems within student populations are as high as 40%, with most students suffering from depression or anxiety, or both. Many respondents in the research expressed the opinion that the number of students with mental health problems was increasing and that the severity of their problems was also increasing. When examining the stress of medical education, the General Professional Education of Physician (GPEP) Report of the Association of American Medical Colleges; recommended increasing the personal development of each student, establishing mentor relationships and placing a greater emphasis on health programs especially stress management techniques, in order to help students cope with the stress of higher education. Students who have realistic expectation in their academic duties, together with a more manageable curriculum, prepared with stress management techniques and high social support would have an advantage in coping (4). In this respect, Medical Faculties should introduce Foundation Courses for new students which provide an overview of what to expect in medical school, as well as lectures on study techniques, stress and time management. To offer a more balanced medical education, talks on what life is like in the faculty, medical history, and the science and art of medicine should also be given. The final goal is to help students understand what is required of them and to adapt as rapidly as possible (7).

## Conclusions

Early detection of psychiatric distress is important to prevent psychiatric morbidity and its unwanted effects on medical students and young doctors. Our results reveals that although a low percentage of Dubai Medical College students reported a significant level of psychiatric distress, however, it should not be underestimated, and actions should be taken to encourage Dubai Medical College students to seek help from psychiatric services for their emotional problems. The risk factors as well as the protective factors must be identified in nation-wide studies to promote mental health of medical students.

## Authors' contributions

JA conceived and designed the evaluation and helped to draft the manuscript. MGA participated in designing the evaluation and performed parts of the statistical analysis. FAB re-evaluated the clinical data and revised the manuscript. AAM evaluated and performed the statistical analysis and revised the manuscript. HA collected the clinical data interpreted them and revised the manuscript. AA re-analyzed the clinical and statistical data and revised the manuscript. All authors read and approved the final manuscript.

## Notes

**Declaration of interest: **None.
